# Offering general pediatric care during the hard times of the 2014 Ebola outbreak: looking back at how many came and how well they fared at a Médecins Sans Frontières referral hospital in rural Sierra Leone

**DOI:** 10.1186/s12887-017-0786-z

**Published:** 2017-01-25

**Authors:** Veerle Hermans, Rony Zachariah, Desalegn Woldeyohannes, Gbessay Saffa, Dauda Kamara, Nimer Ortuno-Gutierrez, Walter Kizito, Marcel Manzi, Petra Alders, Jacob Maikere

**Affiliations:** 1 Médecins Sans Frontières, Brussels Operational Centre, Bo, Sierra Leone; 2 Médecins Sans Frontières, Brussels Operational Centre Brussels, Brussels, Belgium; 3 Aklilu Lemma Institute of Pathobiology, Addis Ababa University, Addis Ababa, Ethiopia; 4 Ministry of Health, Bo, Sierra Leone; 5 Damien Foundation, Conakry, Guinea; 6 Médecins Sans Frontières, Nairobi, Kenya; 7 Médecins Sans Frontières, Brussels Operational Centre, Brussels, Belgium; 8 Vogelzang 41, 2980 Zoersel, Halle Belgium

**Keywords:** SORT-IT, Health care systems, Access to health care, Health worker safety, Infection control

## Abstract

**Background:**

In Bo district, rural Sierra Leone, we assessed the burden of the 2014 Ebola outbreak on under-five consultations at a primary health center and the quality of care for under-15 children at a Médecins Sans Frontières (MSF) referral hospital.

**Methods:**

Retrospective cohort study, comparing a period before (May-October 2013) and during the same period of the Ebola outbreak (2014). Health worker infections occurred at the outbreak peak (October 2014), resulting in hospital closure due to fear of occupational-risk of contracting Ebola. Standardized hospital exit outcomes and case fatality were used to assess quality of care until closure.

**Results:**

A total of 13,658 children under-five, were seen at the primary health center during 2013 compared to 8761 in 2014; a consultation decline of 36%. Of 6497 children seen in the hospital emergency room, during the outbreak, patients coming from within hospital catchment area declined with 38% and there were significantly more self-referrals (80% vs. 61%, *P* < 0.001). During Ebola, 23 children were dead on arrival and the proportion of children in severe clinical status (requiring urgent attention) was higher (74% during Ebola vs. 65% before, *P* < 0.001). Of 5,223 children with available hospital outcomes, unfavorable outcomes (combination of deaths and abandoned) were less than 15% during both periods, which is within the maximum acceptable in-house threshold set by MSF. Case fatality for severe malaria and lower respiratory tract infections (*n* = 3752) were similar (≤15%).

**Conclusions:**

Valuable and good quality pediatric care was being provided in the pediatric hospital during the 2014 Ebola outbreak, but could not be sustained because of hospital closure. Health facility and health worker safety should be tackled as a universal requirement to try to avoid a *déjà-vu.*

## Background

Ebola or Ebola virus disease is a severe disease of humans and primates caused by Ebola viruses [1]. Human-to-human transmission occurs through contact with body fluids of infected individuals. The disease can spread in an exponential manner with high case fatality (30-90%), especially where health systems are fragile [2, 3].

The 2014 Ebola outbreak in West Africa began in Guinea in December 2013 and rapidly spread to Sierra Leone, Liberia and beyond [4]. By June 2016, a total of 28,616 cases out of which 11,310 deaths were reported in the region [5]. By then, a total of 881 health workers have been infected of whom 513 died [5]. Considering the severity of the outbreak, the World Health Organization (WHO) declared the 2014 outbreak an “international public health emergency” [6–8].

Sierra Leone is the worst affected country housing almost half of all cases [5]. Even prior to the Ebola outbreak, the country was struggling to recover from over a decade of civil war, health system limitations and serious health worker shortages [9]. These challenges were exarcerbated by the prolonged nature of the outbreak [2, 10]. For example, several health facilities abruptly shut down because of fear among health workers, compounded by inadequate training and availability of Ebola protective material [10–12]. From a community perspective, fear of contracting Ebola at health facilities, change in health-seeking behaviour and the strict quarantine enforced by the military, could have affected access to care [8].

The Gondama Health Centre and Gondama Referral Hospital are located in a rural district (Bo) of Sierra Leone, badly hit by the Ebola outbreak [13]. The first case in Bo Town was confirmed in June 2014 and there were a total of 482 cases and 175 deaths during the entire outbreak [14]. The Gondama Health Centre is a primary health care facility providing dedicated out-patient consultations for under-five children. On the other hand, the Gondama Referral Hospital is an independent pediatric referral facility, run by Médecins Sans Frontières (MSF), providing emergency room services and admission facilities [15]. There are anecdotal observations that the Ebola outbreak hampered access to both of these facilities. This might have affected under-five consultations at the primary health center. At the hospital, more severe cases were reported in the emergency room, possibly because of delays in patient presentation. The proportion of deaths while on hospital admission, might have also been higher. Such parameters are “proxy” indicators for access-to-care and quality of services and need formal investigation.

A practical problem faced by the two facilities was ensuring infection control and health worker safety in a setting where both health workers and patients moved between different health structures. Unfortunately, in October 2014, five MSF health workers working in Bo district, of which one was working in the Gondama Referral Hospital, were diagnosed with Ebola and two subsequently died. Although these infections were acquired outside the hospital, it resulted in closure of the hospital during the peak of the outbreak.

Looking back, we feel it is important to assess the proxy-indicators of access to and quality of care while reflecting on possible ways-forward for sustaining pediatric services during such epidemics.

One published study from Sierra Leone showed a decline in inpatient admissions and major surgical procedures associated with the Ebola outbreak [16]. A number of other papers from Ebola affected countries in West Africa have reported reduced access to maternal and child health services [17, 18], challenges in prevention and treatment of malaria [19, 20], a drop in routine vaccination [21] and reduced retention in HIV care [22, 23]. No study has as yet, assessed the effect of the Ebola outbreak on both access to and quality of pediatric care. Such information may guide future health system responses (to cope with Ebola) in settings like Sierra Leone where child mortality is high [24].

In Gondama, rural Sierra Leone, we thus aimed to assess the influence of the Ebola outbreak on pediatric consultations at the primary level and the quality of care at the referral hospital.

At the primary level, and for a period before the Ebola outbreak (May to October 2013) and for the same period during the outbreak (2014), we assessed if there was a change in under-five consultation load.

Additionally, at the referral hospital level, for children under 15 years, we compared a) socio-demographic characteristics, clinical severity and emergency room exits and b) among admitted children, their hospital exit outcomes and case fatality.

## Methods

### Study design

Retrospective comparative cohort study.

### Setting

#### General setting

The Republic of Sierra Leone is a poor country in West Africa bordered by two other Ebola affected countries i.e. Guinea and Liberia. The country has a population of about six million and the capital is Freetown [24]. The health infrastructure was crippled by a decade-long civil war that ended in 2002. The country is ranked 11^th^ in infant mortality worldwide [25]. In 2012, a national health sector strategic plan was put in place to make health services readily available, accessible, and affordable, but was hampered because of the serious shortage of qualified health care workers [9]. Even before the Ebola outbreak, there were only 0.2 doctors and 1.7 nurses per 10,000 people, mostly located in urban areas [25].

#### Specific setting

The Gondama primary health center and referral hospital were the two independent study sites. They are located in a rural area, 11 km from Bo Town and within Bo district – 200 km southeast of Freetown. Bo district has a population of about 600,000 and Bo Town is the district capital. Both the primary health center and hospital are located within a proximity of 500 m [15].

### Under five consultations at the gondama primary health center

The *primary health center* is managed by the Ministry of Health (MoH) and was supported by MSF. It provides free out-patient consultations, restricted to children under-five. Those presenting at the primary health center are seen by attending nurses and provided care on an outpatient basis. Children requiring referral care and/or admission were sent to the Gondama referral hospital.

### Emergency room services and hospital admissions at the gondama referral hospital

The *referral hospital* was an independent referral facility and entirely managed by MSF. It provided free secondary level pediatric care since 2003 for children under-15 years. It has 150 beds (but capacity could be increased to 200 according to need) and special wards for neonates, intensive care, malnutrition associated with complications and burns. It also had facilities to provide isolation for suspected Lassa Fever cases [15].

The referral hospital offered emergency room services for children referred from primary health facilities and for self-referrals. Children were first assessed for clinical severity and scored by a nurse in the emergency room according to the South African Triage Scale (SATS) [26]. The scoring allowed emergency staff to designate priority of attention. The clinical grading used in SATS is shown in Table 1.

**Table 1:** Grading of clinical severity and target time to initiate patient management (South African Triage Scale), Gondama hospital, Bo, Sierra Leone

Colour*	Target time	Management
Red	Immediate	Take to resuscitation room for emergency management
Orange	<10 min	Refer for very urgent management
Yellow	<1 h	Refer for urgent management
Green	<4 h	Refer to designated area for non-urgent cases
Blue	<2 h	Refer to doctor for certification

If emergency clinical signs are found, the patient is assigned a *red* priority level and taken straight to the resuscitation area without delay. If no emergency clinical signs are present the patient is assessed for very urgent (*orange*) or urgent (*yellow*) clinical signs. Patients in the *orange* and *yellow* category should be seen within 10 and 60 min respectively. Non-urgent cases are classified as *green* and are to be seen within 4 h. If a patient arrives dead or dies upon arrival, they are considered ‘*blue*’ cases

*adapted from the South African Triage Scale (SATS), Training Manual 2012^26^

Patient information including name, age, sex, address and SATS category was recorded in an emergency room register and a preliminary diagnosis was made. If admission was merited, a patient admission file was opened in which clinical information and hospital exit outcomes were recorded. All hospital exit outcomes are standardized and include discharged, abandoned and died.

### Ebola control measures at primary health center and referral hospital

At both facilities a systematic screening of all patients at facility entry, for suspected Ebola was conducted by MSF. Isolation wards were set up at both facilities. The specific Ebola control measures implemented in both facilities are shown in Table 2.

**Table 2:**
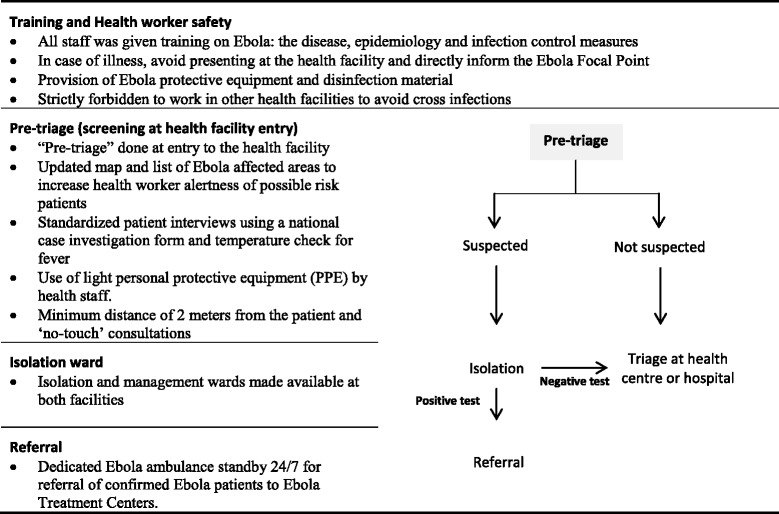
Ebola control measures implemented at the Gondama Health Centre and Referral Hospital, Bo, Sierra Leone (2014)

### Study population and period

The study population included a) at the Gondama primary health center all children (under-five years) presenting for out-patient consultations and b) at the referral hospital level, all children under 15 years presenting at the emergency room and admitted to the hospital wards.

The study period for both facilities included May to October 2013 (before the Ebola outbreak) and during the same period of the Ebola outbreak (May to October 2014).

### Data collection, sources of data and validation

Aggregate data on numbers of consultations at the Gondama Primary health center was collected from health center data sheets on a weekly basis. Data were encoded weekly into Microsoft Excel.

At the referral hospital, data on demographic characteristics, clinical severity, exit outcomes was sourced from dedicated emergency room and hospital ward registers. Case fatality was assessed for the two main morbidities: severe malaria and lower respiratory tract infections. Data were encoded daily into two separate excel (MicroSoft Excel) databases. Data quality was assured by cross-checking data sheets with registers for consistency.

### Statistical analysis

A total of 3436 and 1787 children were included in the study for the years 2013 and 2014, respectively; making a total of 5223 children for the referral hospital sufficiently large to perform reliable statistical analysis. Descriptive statistics were used to express results and differences between groups were assessed using the Chi-square (*X* ^2^) test . A two-sample Independent *t*-test on difference between means was performed on the SATS variable and Relative Risks were used to compare hospital exit outcomes. The level of significance was set at *P* ≤ 0.05 and 95% confidence intervals were used. Analysis was performed using EpiData Analysis software (version 2.2.2.182, EpiData Association, Odense, Denmark).

## Results

### Pediatric consultations at the primary health care level

A total of 13,658 children under-five, were seen before the Ebola outbreak compared to 8,761 during the outbreak; a decline of 4,897 (36%) outpatient consultations.

### Characteristics, clinical severity and emergency room exits at the referral hospital level

Table 3 shows the socio-demographic characteristics of these children, their clinical severity and emergency room outcomes. A total of 6,497 children were seen in the hospital emergency room during 2013 and 2014. Age distribution was similar for the two periods with 90% of the children being under-five (range: 1 day-15 years).

**Table 3:** Characteristics, clinical severity and emergency room exits of children who arrived at the Referral hospital, Bo Sierra Leone

Variable	2013 *n* (%)	2014 *n* (%)	*P*-value^1^
Total	3953	2544	
Gender			
Male	2120 (54)	1398 (55)	0.3
Female	1833 (46)	1146 (45)	
Age			
< 5	3531 (89)	2250 (88)	0.3
5-15	422 (11)	294 (12)	
Origin^2^			
In catchment area	3700 (94)	2290 (90)	<0.001
Outside catchment area	253 (6)	254 (10)	
Referred by			
Self-referral	2419 (61)	2044 (80)	<0.001
Primary health facility	1527 (39)	496 (19)	<0.001
Governmental hospital	7 (<1)	4 (<1)	0.8
Clinical severity (SATS^3^)			
Red	1232 (31)	936 (37)	<0.001
Orange	1363 (34)	935 (37)	
Yellow	919 (23)	487 (19)	
Green	439 (11)	163 (6)	
Blue^4^	0	23 (1)	
Emergency room exits			
Admitted to pediatric ward	3652 (92)	2130 (84)	<0.001
Died^5^	43 (1)	51 (2)	0.01
Referred to other health facility	45 (1)	56 (2)	0.01
Discharged with medical consent	212 (5)	301 (12)	<0.001
Discharged without medical consent	1 (<1)	6 (<1)	0.01

^1^χ^2^ test for categorical variables and two-sample independent *t*-test on difference between means for SATS variables

^2^Geographic catchment area of the Gondama Referral Hospital

^3^SATS: Clinical severity based on the South African Triage Scale, see Table 1

^4^Arrived dead or died upon arrival

^5^Including patients in the ‘*blue*’ SATS category

During the Ebola outbreak the number of patients coming from within the hospital catchment area declined by 1410 (38%), while the number of patients from outside the catchment area remained constant. In addition, more self-referrals were observed (80% vs. 61%).

During Ebola, 23 children were dead on arrival at the emergency room; no such deaths were reported prior to the outbreak. Fifteen of these children died of severe malaria, other causes of death were severe malnutrition (*n* = 2), sepsis (*n* = 1), hypoglycemia (*n* = 1) and unknown (*n* = 4). The combined proportion of children requiring immediate (SATS red category) and urgent attention (SATS orange category) was also significantly higher during the Ebola outbreak compared to the prior period (74% vs. 65%) In 2014 several training sessions were done to strengthen the adherence to admission criteria which might explain the observation that fewer patients were admitted in 2014 (84% compared to 92% in 2013).

### Hospital exit outcomes and case fatality

Of 5782 children admitted from the emergency room to the hospital wards, exit outcomes were unrecorded for 559 (10%) children, who were excluded. The hospital exit outcomes for 2013 and 2014 of the remaining 5223 are shown in Table 4. Significantly more children (87%) were successfully discharged during the Ebola outbreak compared to the preceding year. Unfavorable hospital exit outcomes (combination of deaths and abandoned) were less than 15% during both periods, which is within the maximum acceptable in-house threshold set by MSF [27].

**Table 4:** Exit outcomes at the Referral hospital, Bo, Sierra Leone

	2013 *n* (%)	2014 *n* (%)	Relative Risk (95% CI^1^)	*P*-value^2^
Total	3436	1787		
Recovered and discharged	2770 (81)	1560 (87)	1.1 [1.1-1.1]	<0.001
Unfavorable outcomes				
Total	329 (10)	199 (11)	1.2 [1.0-1.4]	0.08
Died	245	174		
Abandoned^3^	84	25		
Referred out^4^	337 (10)	28 (2)	0.2 [0.1-0.2]	<0.001

^1^95% CI = 95% Confidence Interval

^2^χ2 test

^3^Abandoned: discharged without medical consent, patient leaves the hospital against medical advice

^4^refered to other health facility for further care, with final outcome unknown

More children (10%) were also referred out to other facilities prior to the Ebola outbreak than during (2%).

Case fatality for severe malaria and lower respiratory tract infections is presented in Table 5. Of a total of 5,223 hospital exits, 3752 (72%) children were diagnosed with either one of these two conditions. There was no significant difference in case fatality for these morbidities.

**Table 5:** Number of cases and case fatality for main morbidities by exit diagnosis at the Referral hospital

	2013		2014	
	Total	Deaths *n* (%)^1^	Total	Deaths *n* (%)^1^
Total	2458		1294	
Severe malaria	2239	134 (6)	1127	80 (7)
Lower respiratory tract infections	219	27 (12)	167	25 (15)

^1^ *P*-values for *χ*2 test > 0.05

## Discussion

This is a first study from West Africa assessing the influence of the 2014 Ebola outbreak on both access to and quality of pediatric care at both primary and hospital level. A considerable decline in outpatient consultations was seen at the primary level while at the referral hospital, more children presented from far-and-wide and in severe clinical status. Encouragingly, once admitted to the hospital wards, quality of care as assessed by hospital exit outcomes and case fatality were within acceptable standards.

This study highlights the added value of upholding pediatric health services during Ebola outbreaks in a country with high child mortality [24]. However, when health care worker and patient safety cannot be ensured, it is justified to close a hospital until both, quality of care and a safe working environment, can be granted. Unfortunately, the resulting “care vacuum” is likely to have exacerbated the predicament of vulnerable children in this rural setting.

The study strengths are that we included data from a well-monitored primary health center and referral hospital, at the district level in rural Sierra Leone; the study sample was large, data were entered by dedicated data entry clerks and supervised by a data manager; clinical severity was scored using the standardized SATS system [26]; hospital exit outcomes were cross-checked with patient records; and we followed STROBE guidelines in reporting of observational studies [28].

A study limitation is that we did not have hospital exit outcomes for 10% of children admitted from the emergency room to the hospital wards. This was because the neonatology database did not have unique identity coding allowing linkage with emergency room registers. We thus excluded all neonates from the hospital exit analysis. The importance of addressing this operational problem has been noted. In addition, the findings of this study cannot be generalized for pediatric care in rural health facilities in Sierra Leone. Presumably other health facilities might not have been able to uphold this quality of care due to infections and deaths among health care workers, no availability of Ebola protective material and fear of treating patients [5, 10–12]. A retrospective study using data from different rural health facilities might shed more light on this matter.

There are a number of important findings from this study. First, we observed a 36% drop in under-five consultations at the primary level. This is similar to what has been reported from Guinea, Liberia and Sierra Leone. It shows that utilization of health services including child health services were compromised during the Ebola outbreak [12, 18–21]. For example, in Guinea, where the impact of the Ebola outbreak on primary care outpatient consultations was assessed, consultations dropped by 30% [18]. Similarly, utilization of HIV-testing services dropped by 40% during the outbreak compared to the pre-outbreak period [22]. Such declines may be due to hardships faced in reaching the health facility and/or community fear that health facilities are unsafe places to visit [11].

Second, in the emergency room of the referral hospital, there were more children from outside the geographic catchment area and more self-referrals. There may be two possible reasons; closure of many health facilities within and outside the district; and decline in economic activity related to the strict Ebola quarantine (including roadblocks) affecting “ability to pay”. Health seeking behavior favoring free-health-care may have taken precedent.

Third, during the Ebola outbreak, 23 children were actually dead by the time they arrived at the hospital and the proportion requiring urgent attention, was considerably higher. These delays in presentation may be due to limited public transport, roadblocks and fear of forced quarantine. A re-assuring and encouraging finding was that unfavorable hospital exit outcomes (deaths and abandoned) remained below the desired MSF threshold of 15%. The observation that significantly more children were successfully discharged during the Ebola outbreak can also be explained by the fact that several facilities where these children were referred to in 2013 already closed or were unsafe to refer patients. The patients were therefore managed at the referral hospital until discharge. The over-riding message is that irrespective of clinical severity, once admitted to the hospital wards, children fared well in this referral MSF hospital. The fact that case fatality for severe malaria and lower respiratory tract infections also remained similar during the two periods adds value to such judgement.

In retrospect, what was most discouraging is that despite the valuable service offered by the referral hospital, it still ended up being closed for fear of health worker safety. *Looking back, what could we have done better to avoid this eventuality?*

Although it was strictly forbidden for health workers to juggle work between health facilities in order to prevent Ebola cross-infection, this was practically impossible. It is understandable that health workers who have dependents and added financial responsibilities will seek additional revenue by working elsewhere or providing home based care. Both these activities expose health workers to Ebola outside their mother health facility. This makes health facilities and health workers inter-dependable in terms of occupational-risk.

Three clear recommendations come to light. First, strict infection control and health worker protection need to be addressed as a “universal measure” applicable to all health facilities in an Ebola affected region. Second, triage for suspected Ebola should not only be patient-centered (as it was), but also “health-worker” centered. Third, when this becomes a reality, front-line health workers should be prioritized for Ebola preventive vaccination [29].

In the meantime, the antidote to upholding health services is health worker safety and universal infection control [30]. We thus reiterate our urgent earlier call to the WHO and Governments of African countries to establish dedicated umbrella units to ensure health worker and facility safety (occupational health) [31, 32].

## Conclusion

In conclusion, during an Ebola outbreak in rural Sierra Leone, valuable and good quality pediatric care was being provided, but it could not be sustained due to issues of health facility and health worker safety. Serious attention will be needed in this regard to avoid a *déjà-vu.*

## Abbreviations

CI: Confidence Interval
MoH: Ministry of Health
MSF: Médecins Sans Frontières
SATS: South African Triage Scale
WHO: World Health Organization
